# Serum uric acid as a predictor of mortality in patients with stroke: results from National Health and Nutrition Examination Survey 2007–2016

**DOI:** 10.3389/fneur.2024.1383300

**Published:** 2024-06-26

**Authors:** Xinyu Tong, Chuxin Lyu, Minjie Guo, Jianxiong Gu, Yichun Zhao

**Affiliations:** ^1^Department of Neurology, Wuxi Traditional Chinese Medicine Hospital, Wuxi, China; ^2^First Clinical Medical School, Nanjing University of Chinese Medicine, Nanjing, China

**Keywords:** serum uric acid, stroke, mortality, NHANES, Cox regression

## Abstract

**Objective:**

This research endeavors to explore the relationship between serum uric acid (SUA) concentration and all-cause mortality in stroke patients.

**Methods:**

We undertook a cross-sectional analysis utilizing data derived from the National Health and Nutrition Examination Survey (NHANES) spanning 2007 to 2016. The concentrations of SUA served as the independent variable, while the dependent variable was defined as all-cause mortality in stroke patients. The quartile method was utilized to classify uric acid levels into four distinct categories. Subsequently, three models were developed, and Cox proportional hazards regression was used to assess the effect of varying uric acid concentrations on the risk of all-cause mortality among stroke patients.

**Results:**

The study included a total of 10,805 participants, of whom 395 were stroke patients. Among all populations, the group with elevated levels of uric acid (Q4) exhibited a significant association with the overall mortality risk among stroke patients in all three models (model 1 *p* < 0.001, model 2 *p* < 0.001, model 3 *p* < 0.001). In the male population, there was no significant correlation observed between uric acid levels and the overall mortality risk among stroke patients in model 3 (Q2 *p* = 0.8, Q3 *p* = 0.2, Q4 *p* = 0.2). However, within the female population, individuals with high uric acid levels (Q4) demonstrated a noteworthy association with the overall mortality risk among stroke patients across all three models (model 1 *p* < 0.001, model 2 *p* < 0.001, model 3 *p* < 0.001).

**Conclusion:**

This cross-sectional investigation reveals a significant correlation between SUA levels and all-cause mortality in stroke patients, with a noticeable trend observed among females. Consequently, SUA may serve as a promising biomarker for assessing the prognosis of individuals affected by stroke.

## Introduction

Stroke, a term encompassing sudden localized or diffuse neurological deficits, is attributed to the disturbance of blood circulation. Sometimes stroke is covert. According to the Global Burden of Diseases, Injuries and Risk Factors Study (GBD) 2017, stroke is ranked as the third leading cause of mortality and disability, quantified by disability-adjusted life years (DALY). Furthermore, it is the second most significant contributor to death and disability (1, 2). In 2017, the global incidence of acute ischemic strokes was approximately 950 per 100,000 individuals (3).

Following a significant long cessation of oxygen supply resulting from blood inflow or outflow disturbance, a sequence of cascading events (4) is initiated, which includes ATP depletion, alterations in sodium, potassium, and calcium ion concentrations, an increase in lactic acid, acidosis, the accumulation of oxygen free radicals, cell edema, and proteolysis. These processes ultimately result in cell death and neurological deficits. Prior research has identified numerous contributing factors to these neurological deficits after a stroke. It has been found that hypertension, suboptimal blood glucose control, smoking, alcohol consumption, and other unhealthy lifestyle habits significantly influence neurological deficits following a stroke (5–9). Serum uric acid (SUA) serves as a prevalent index in serological tests. Its impact on blood pressure and renal function is notable, particularly due to its role in generating oxidative stress via xanthine oxidase. This enzyme binds with endothelial cells, thereby inhibiting the activity of nitric oxide (NO), which results in vascular damage. Inflammation and damage of blood vessels lead to atherosclerosis, thereby promoting the occurrence and development of stroke.

Several prior meta-analyses have demonstrated that hyperuricemia marginally elevates the risk of stroke morbidity and mortality (10, 11). However, contrasting findings suggest the potential beneficial effects of SUA on the central nervous system (12, 13). Reactive oxygen species, induced by ischemia/reperfusion injury, play a significant role in neuronal cell death. Consequently, the antioxidant properties of SUA may be advantageous for neuronal survival. Currently, only a handful of small-scale studies have indicated that a reduction in serum uric acid (SUA) levels is independently linked to adverse outcomes following acute ischemic stroke (14). Therefore, further research is required to substantiate whether SUA levels can effectively influence the prognosis of stroke patients.

However, limited research has been conducted to explore the relationship between SUA levels and all-cause mortality. The optimal range of SUA levels that could prevent death remains ambiguous, necessitating substantial evidence from the general population to fill these knowledge gaps. Consequently, this study will utilize the public NHANES database, which boasts a large sample size. We conducted a cross-sectional study utilizing pooled data from the NHANES spanning 2007 to 2016, aiming to elucidate the specific association between SUA levels and all-cause mortality in patients with stroke.

## Methods

### Population, design

The NHANES serves as a nationally representative survey of the US population, providing comprehensive data on nutrition and health within the general US populace. The survey data are publicly accessible to researchers and users. The National Center for Health Statistics (NCHS) conducts the collection of these data on a biennial basis (15). The National Health Care and Nutrition Evaluation System of methods and data collection work is in front of the content for the instructions and can be in the national health care. For further reference, this information can be accessed through the National Health Care and Nutrition Evaluation System’s consultation website^1^ (15). The NCHS Research Ethics Review Board has granted approval for the survey protocol, and all participants have furnished written informed consent.

**Figure 1:**
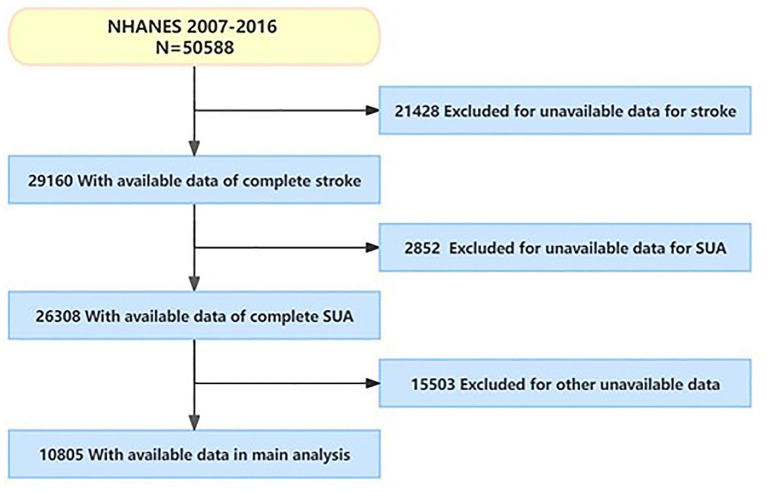
Flowchart of research subject screening.

We conducted a cross-sectional study using pooled data from NHANES between 2007 and 2016. A total of 50,588 participants completed the visit in the current survey cycle. Self-reported history of stroke was used as an outcome measure. Stroke histories were collected using structured questionnaire administered by trained interviewers at home, and finally, 10,805 individuals were included (Figure 1). Patients were segregated into two groups: the stroke group and the non-stroke group, based on their respective diagnoses of stroke.

### Methodologies for the identification of SUA

The timed end-point method was employed to quantify the concentration of uric acid in serum, plasma, or urine samples. Uric acid undergoes oxidation by uricase, resulting in the formation of allantoin and hydrogen peroxide. Subsequently, hydrogen peroxide interacts with 4-amino antipyrine (4-AAP) and 3,5-dichloro-2-hydroxybenzenesulfonate (DCHBS), a process catalyzed by peroxidase, yielding colored products. The system tracked changes in absorbance at 520 nm at consistent time intervals. The variation in absorbance is directly proportional to the uric acid concentration within the sample. Of the total participants, 67,530 completed the test, while 5,610 did not. We categorized SUA levels into four distinct quartiles: ≤267.7 μmol/L (group 1), 273.6–321.2 μmol/L (group 2), 327.1–374.7 μmol/L (group 3), and >380.7 μmol/L (group 4). All laboratory measurement data can be accessed publicly on the NHANES website.

### Covariates

Categorical variables were included as covariates in the analyses: gender, educational level, poverty index, hypertension, diabetes, leisure physical activity and kidney disease. Continuous variables were included in the analyses: age, high-density lipoprotein, low-density lipoprotein, triglycerides, body mass index sedentary hours and glomerular filtration rate (GFR). Educational attainment was categorized into three levels: high school or less, college, and college graduate. The Household Monthly Poverty Index compares household income to the poverty line using the poverty line to evaluate income levels and eligibility for federal assistance programs. The rates were categorized as <10, 130 to 185% and >185%. Blood pressure values were derived as an average from multiple measurements conducted at the mobile examination center. The medical definition of hypertension was established when systolic blood pressure (SBP) ≥ 140 mmHg and/or diastolic blood pressure (DBP) ≥90 mmHg, as measured in repeated office blood pressure assessments (16). Diabetes was delineated by a glycosylated hemoglobin A1c (HbA1c) value exceeding 6.5%, fasting blood glucose (Glu) levels surpassing 7.0 mmol/L, current utilization of antidiabetic medications, or a prior history of diabetes (17). Leisure physical activity was determined by the frequency of engaging in moderate-intensity exercise, fitness routines, or recreational activities for at least 10 min per week. Kidney disease refers specifically to disorders affecting the kidneys, excluding conditions such as kidney stones, bladder infections, or incontinence. The sedentary index was ascertained by counting the number of sedentary minutes per day. LDL-C, HDL-C, triglycerides, and GFR were ascertained through laboratory results.

We also conducted an analysis on the correlation between different levels of uric acid and the overall risk of mortality in male and female stroke patients separately.

### Statistical methods

In this research, we employed restricted cubic splines (RCS) to conduct a quantitative analysis of the relationship between individual SUA levels and mortality risk in stroke patients. The subsequent analysis involved a quantitative assessment of the association between uric acid concentration and mortality risk in male and female stroke patients, separately. The Cox proportional hazards models were employed to ascertain the hazard ratio (HR) and 95% confidence interval (CI) for the correlation between the SUA index and mortality. Three distinct models were fitted: model 1 was unadjusted for covariates; model 2 was adjusted for gender, kidney disease, and GFR, and model 3 was further refined by adjusting for serum low-density lipoprotein, high-density lipoprotein, triglycerides, body mass index (BMI), and the number of sedentary minutes per day. Stratified analyses were conducted based on sex, age (categorized as <60 years or ≥ 0 years), the presence or absence of hypertension, and the existence or nonexistence of diabetes. In the statistical analysis process, sample weights were determined following the NHANES data analysis guidelines. A two-tailed *p* < 0.05 was considered statistically significant. All statistical analyses were performed using R version 3.4.3.

## Results

The study incorporated a total of 10,805 subjects, comprising 395 in the stroke group and 10,410 in the control group. Of these participants, 5,171 (48%) were male, with a *p*-value of 0.2. The mean age of the cohort was 48 ± 17 years (*p* < 0.001). Notable differences were observed between the stroke and control groups concerning factors such as race, education, marital status, poverty index, hypertension, diabetes, low-density lipoprotein levels, SUA concentration, and leisure-time physical activity. However, no statistically significant disparities were found in BMI, sedentary index, triglycerides, and high-density lipoprotein levels (Table 1).

**Table 1:** Characteristics of participants based on their respective diagnoses of stroke.

Characteristic	Overall, *N* = 10,805^a^	Stroke, *N* = 395^b^	Non-stroke, *N* = 10,410^b^	*p*-value^b^
Sex				0.13
Male	5,171 (48%)	184 (42%)	4,987 (48%)	
Age (years)	48 (17)	64 (14)	47 (17)	<0.001
Educational level				<0.001
Less than 9th grade	1,110 (5.6%)	55 (8.6%)	1,055 (5.5%)	
9–11th grade	1,552 (11%)	92 (21%)	1,460 (11%)	
High school grad/GED or equivalent	2,405 (21%)	99 (28%)	2,306 (21%)	
Some college or AA degree	3,092 (31%)	96 (25%)	2,996 (31%)	
College graduate or above	2,646 (31%)	53 (18%)	2,593 (32%)	
Ratio of family income to poverty	2.97 (1.65)	2.40 (1.53)	2.98 (1.65)	<0.001
Hypertension				<0.001
Yes	3,848 (32%)	295 (71%)	3,553 (31%)	
Diabetes				<0.001
Yes	1,361 (9.5%)	126 (28%)	1,235 (9.0%)	
HDL-cholesterol (mmol/L)	1.42 (0.43)	1.40 (0.47)	1.42 (0.43)	0.3
LDL-cholesterol (mmol/L)	2.96 (0.91)	2.73 (0.99)	2.97 (0.91)	<0.001
Uric acid (μmol/L)	325 (82)	347 (90)	324 (82)	<0.001
Triglyceride (mmol/L)	1.33 (0.75)	1.38 (0.74)	1.33 (0.75)	0.13
Body mass index (kg/m^2^)	29 (7)	30 (7)	29 (7)	0.3
Moderate recreational activities (≥10 min)				0.001
Yes	4,335 (45%)	110 (34%)	4,225 (45%)	
Kidney disease				<0.001
Yes	290 (2.1%)	35 (7.7%)	255 (1.9%)	
GFR (mL/min)				
	89 (30)	71 (30)	90 (30)	<0.001

a *n* (unweighted) (%); mean (SD).

b The chi-squared test was conducted with Rao & Scott’s second-order correction, while the Wilcoxon rank-sum test was employed for complex survey samples.

We developed three multivariate Cox regression models, examining the correlation between SUA levels and all-cause mortality in stroke patients (Table 2): model 1, unadjusted for any covariates and using Q1 as the reference, yielded hazard ratios [HRs (95% CI)] for all-cause mortality in the remaining three groups of 1.19 (0.94, 1.49), 1.25 (1.01, 1.54), and 1.77 (1.44, 2.18), respectively (*p* = 0.15, *p* = 0.040, *p* < 0.001). Model 2, adjusted for gender, kidney disease and GFR, using Q1 as the reference, yielded hazard ratios [HRs (95% CI)] of 1.17 (0.93, 1.47), 1.23 (0.99, 1.53), and 1.68 (1.36, 2.08) for all-cause mortality in the remaining three groups, respectively (*p* = 0.2, *p* = 0.065, *p* < 0.001). Model 3, which was further adjusted for serum low-density lipoprotein, high-density lipoprotein, triglycerides, BMI, and the number of sedentary minutes per day, used the Q1 group as a reference. The hazard ratios [HRs (95% CI)] for all-cause mortality in the remaining three groups were found to be 1.19 (0.93, 1.52), 1.33 (1.05, 1.70), and 1.80 (1.41, 2.29), respectively (*p* = 0.2, *p* = 0.020, *p* < 0.001). We developed three multivariate Cox regression models to investigate the association between serum uric acid (SUA) levels and all-cause mortality in male stroke patients (Table 3). Model 1, which was not adjusted for any covariates and used Q1 as the reference group, yielded hazard ratios [HRs (95% CI)] of 0.94 (0.61, 1.44), 1.26 (0.81, 1.95), and 1.22 (0.85, 1.76) for all-cause mortality in the remaining three groups, respectively, (*p* = 0.8, *p* = 0.3, *p* = 0.3). The second model, adjusted for kidney disease and GFR, with Q1 as the reference group, yielded hazard ratios [HRs (95% CI)] of 0.92 (0.60, 1.43), 1.26 (0.81, 1.95), and 1.17 (0.81, 1.95) for all-cause mortality in the remaining three groups, respectively (*p* = 0.7, *p* = 0.3, *p* = 0.3). Model 3 further adjusted for serum low-density lipoprotein levels, high-density lipoprotein levels, triglycerides levels, BMI, and sedentary minutes per day using the Q1 group as a reference. The hazard ratios [HRs (95% CI)] for all-cause mortality in the remaining three groups were found to be 0.96 (0.62, 1.48), 1.37 (0.83, 2.25), and 1.35 (0.86, 2.12), respectively (*p* = 0.8, *p* = 0.2, *p* = 0.2). In these three models considered, a significant association was observed between SUA levels across four distinct groups and all-cause mortality in stroke patients. We also developed three multivariate Cox regression models, examining the correlation between SUA levels and all-cause mortality in female stroke patients (Table 4): model 1, which was not adjusted for any covariates and used Q1 as the reference group, yielded hazard ratios [HRs (95% CI)] of 1.30 (0.97, 1.75), 1.34 (1.00, 1.78), and 2.01 (1.47, 2.75) for all-cause mortality in the remaining three groups, respectively, (*p* = 0.080, *p* = 0.049, *p* < 0.001). The second model, adjusted for kidney disease and GFR with Q1 as the reference group, yielded hazard ratios (HRs) of 1.27 (95% CI: 0.97–1.67), 1.22 (95% CI, 0.94–1.59), and 1.89 (95% CI, 1.43–2.49) for all-cause mortality in the remaining three groups, respectively (*p* = 0.086, *p* = 0.14, *p* < 0.001). Additionally, model 3 further adjusted for serum low-density lipoprotein, high-density lipoprotein, triglycerides, BMI, and sedentary minutes per day while using the Q1 group as a reference. The hazard ratios [HRs (95% CI)] for all-cause mortality in the remaining three groups were determined to be 1.30 (0.97, 1.75), 1.34 (1.00, 1.78), and 2.01 (1.47, 2.75), respectively (*p* = 0.080, *p* = 0.049, *p* < 0.001). Notably, a significant association was observed between serum uric acid levels across the four distinct groups and all-cause mortality in stroke patients in the three models considered. Among all populations, the group with elevated levels of uric acid (Q4) exhibited a significant correlation with the overall mortality risk among stroke patients. In the male population, there was no significant association between serum uric acid levels and the overall mortality risk among stroke patients; however, in the female population, the group with high uric acid levels (Q4) demonstrated a significant correlation with the overall mortality risk among stroke patients. Additionally, we employed three multivariate Cox regression models to investigate the relationship between serum uric acid (SUA) levels and cardiovascular and cerebrovascular mortality rates in stroke patients (refer to Table 2). The correlation was found to be statistically significant for both cardiovascular and cerebrovascular mortality rates but not for others. According to findings from the URRAH study (18), SUA was confirmed as an independent risk marker for stroke events, establishing that >284.91 μmol/L served as an effective predictive cutoff value. Based on this cutoff value from the URRAH study, participants were divided into G1 group (SUA ≤284.91 μmol/L) and G2 group (SUA >284.91 μmol/L). We further utilized these three multivariate Cox regression models to examine the association between SUA levels and overall mortality rate among stroke patients using this cutoff value from URRAH study (see Table 5): model 1 did not adjust for any covariates while considering G1 as reference; HRs and corresponding 95% confidence intervals indicated that G2’s overall mortality rate was significantly higher at 1.35 (1.15–1.59) (*p* < 0.001). The second model, adjusted for kidney disease and GFR with G1 as the reference group, revealed hazard ratios [HRs (95% CI)] of 1.31 (1.10, 1.55) for all-cause mortality in G2 (*p* = 0.002). In the third model, which additionally accounted for serum low-density lipoprotein, high-density lipoprotein, triglycerides, BMI, and sedentary minutes per day while using the G1 group as a reference group, the hazard ratios [HRs (95% CI)] for all-cause mortality in G2 were found to be 1.35 (1.12, 1.62) (*p* = 0.002). Across these three models considered here, a significant association was observed between SUA levels across four distinct groups and all-cause mortality in stroke patients.

**Table 2:** Multivariate Cox regression analysis of the SUA ratio quintiles and all-cause mortality, cardiovascular mortality, and cerebrovascular mortality.

All-cause mortality	Model 1 HR (95% CI), *p*-value	Model 2 HR (95% CI), *p*-value	Model 3 HR (95% CI), *p*-value
*SUA ratio quintiles*			
Q1	Ref	Ref	Ref
Q2	1.19 (0.94, 1.49), 0.15	1.17 (0.93, 1.47), 0.2	1.19 (0.93, 1.52), 0.2
Q3	1.25 (1.01, 1.54), 0.040	1.23 (0.99, 1.53), 0.065	1.33 (1.05, 1.70), 0.020
Q4	1.77 (1.44, 2.18), <0.001	1.68 (1.36, 2.08), <0.001	1.80 (1.41, 2.29), <0.001
*p* for trend	<0.001	<0.001	<0.001
*Cardiovascular mortality*			
Q1	Ref	Ref	Ref
Q2	1.16 (0.70, 1.91), 0.6	1.15 (0.70, 1.90), 0.6	1.03 (0.63, 1.66), >0.9
Q3	1.01 (0.65, 1.58), >0.9	1.01 (0.65, 1.57), >0.9	0.95 (0.59, 1.53), 0.8
Q4	1.48 (0.92, 2.36), 0.11	1.43 (0.87, 2.35), 0.2	1.27 (0.76, 2.11), 0.4
*p* for trend	0.140	0.196	0.413
*Cerebrovascular mortality*			
Q1	Ref	Ref	Ref
Q2	1.16 (0.47, 2.85), 0.7	1.15 (0.47, 2.81), 0.8	1.05 (0.41, 2.69), >0.9
Q3	1.60 (0.64, 3.98), 0.3	1.57 (0.62, 3.95), 0.3	2.16 (0.97, 4.82), 0.060
Q4	1.74 (0.77, 3.94), 0.2	1.59 (0.70, 3.60), 0.3	2.22 (1.05, 4.72), 0.037
*p* for trend	0.156	0.231	0.016

**Table 3:** In male patients, multivariate Cox regression analysis of the SUA ratio quintiles and all-cause mortality, cardiovascular mortality, and cerebrovascular mortality.

All-cause mortality	Model 1 HR (95% CI), *p*-value	Model 2 HR (95% CI), *p*-value	Model 3 HR (95% CI), *p*-value
*SUA ratio quintiles*			
Q1	Ref	Ref	Ref
Q2	0.94 (0.61, 1.44), 0.8	0.92 (0.60, 1.43), 0.7	0.96 (0.62, 1.48), 0.8
Q3	1.26 (0.81, 1.95), 0.3	1.26 (0.81, 1.95), 0.3	1.37 (0.83, 2.25), 0.2
Q4	1.22 (0.85, 1.76), 0.3	1.17 (0.81, 1.95), 0.3	1.35 (0.86, 2.12), 0.2
*p* for trend	0.126	0.188	0.110

**Table 4:** In female patients, multivariate Cox regression analysis of the SUA ratio quintiles and all-cause mortality, cardiovascular mortality, and cerebrovascular mortality.

All-cause mortality	Model 1 HR (95% CI), *p*-value	Model 2 HR (95% CI), *p*-value	Model 3 HR (95% CI), *p*-value
*SUA ratio quintiles*			
Q1	Ref	Ref	Ref
Q2	1.30 (0.97, 1.75), 0.080	1.27 (0.97, 1.67), 0.086	1.30 (0.97, 1.75), 0.080
Q3	1.34 (1.00, 1.78), 0.049	1.22 (0.94, 1.59), 0.14	1.34 (1.00, 1.78), 0.049
Q4	2.01 (1.47, 2. 75), <0.001	1.89 (1.43, 2.49), <0.001	2.01 (1.47, 2.75), <0.001
*p* for trend	<0.001	<0.001	<0.001

**Table 5:** Multivariate Cox regression analysis of the cutoff values of SUA in the URRAH study and all-cause mortality, cardiovascular mortality, and cerebrovascular mortality.

All-cause mortality	Model 1 HR (95% CI), *p*-value	Model 2 HR (95% CI), *p*-value	Model 3 HR (95% CI), *p*-value
*SUA ratio quintiles*			
G1	Ref	Ref	Ref
G2	1.35 (1.15, 1.59), <0.001	1.31 (1.10, 1.55), 0.002	1.35 (1.12, 1.62), 0.002

Cardiovascular mortality	Model 1 HR (95% CI), *p*-value	Model 2 HR (95% CI), *p*-value	Model 3 HR (95% CI), *p*-value
*SUA ratio quintiles*			
G1	Ref	Ref	Ref
G2	1.22 (0.87, 1.73), 0.2	1.21 (0.85, 1.71), 0.3	1.15 (0.83, 1.60), 0.4

Cerebrovascular mortality	Model 1 HR (95% CI), *p*-value	Model 2 HR (95% CI), *p*-value	Model 3 HR (95% CI), *p*-value
*SUA ratio quintiles*			
G1	Ref	Ref	Ref
G2	1.62 (0.82, 3.19), 0.2	1.53 (0.77, 3.07), 0.2	1.94 (1.02, 3.69), 0.043

**Figure 2:**
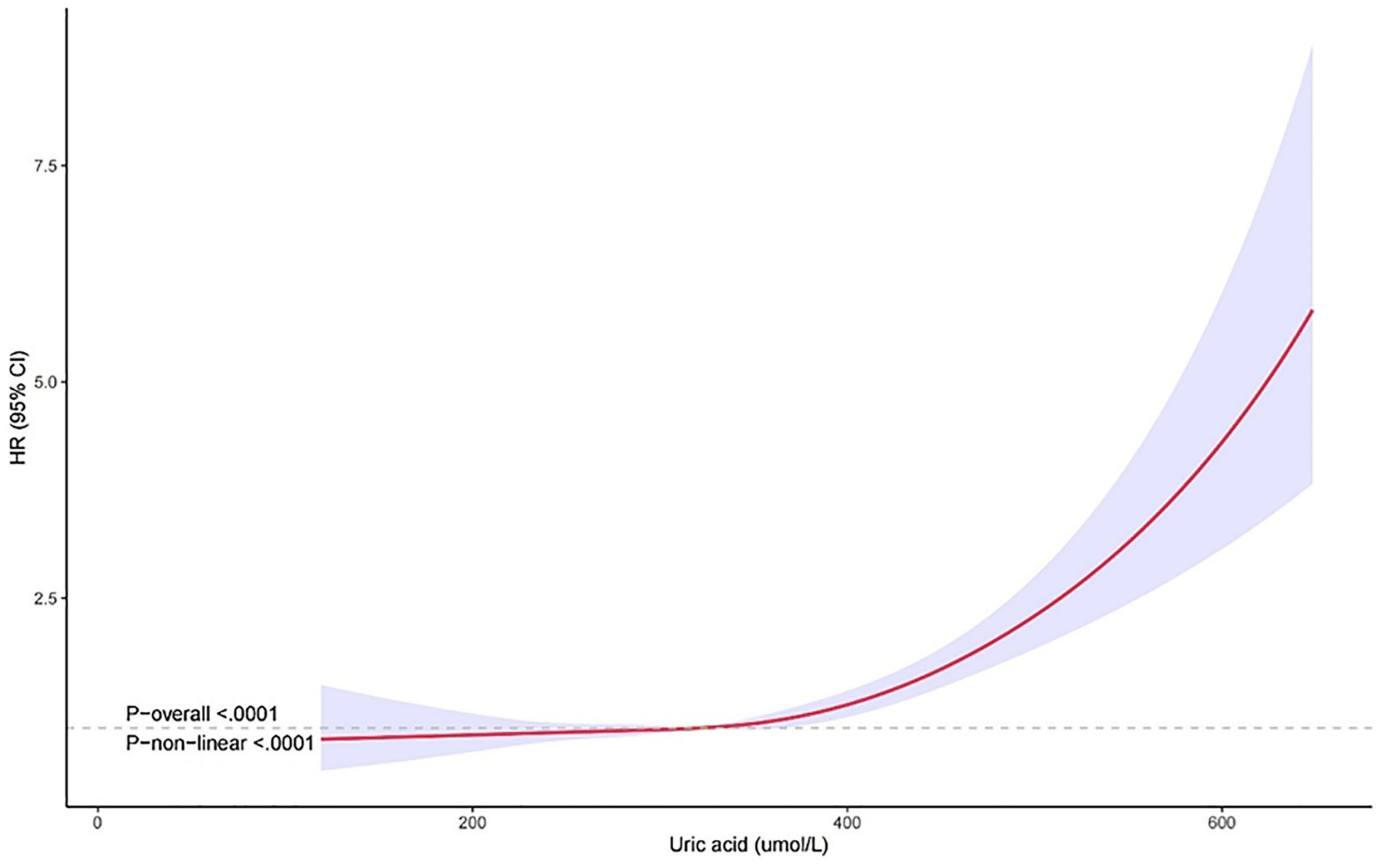
Nonlinear analysis of all-cause mortality and SUA ratio quintiles in stroke patients.

**Figure 3:**
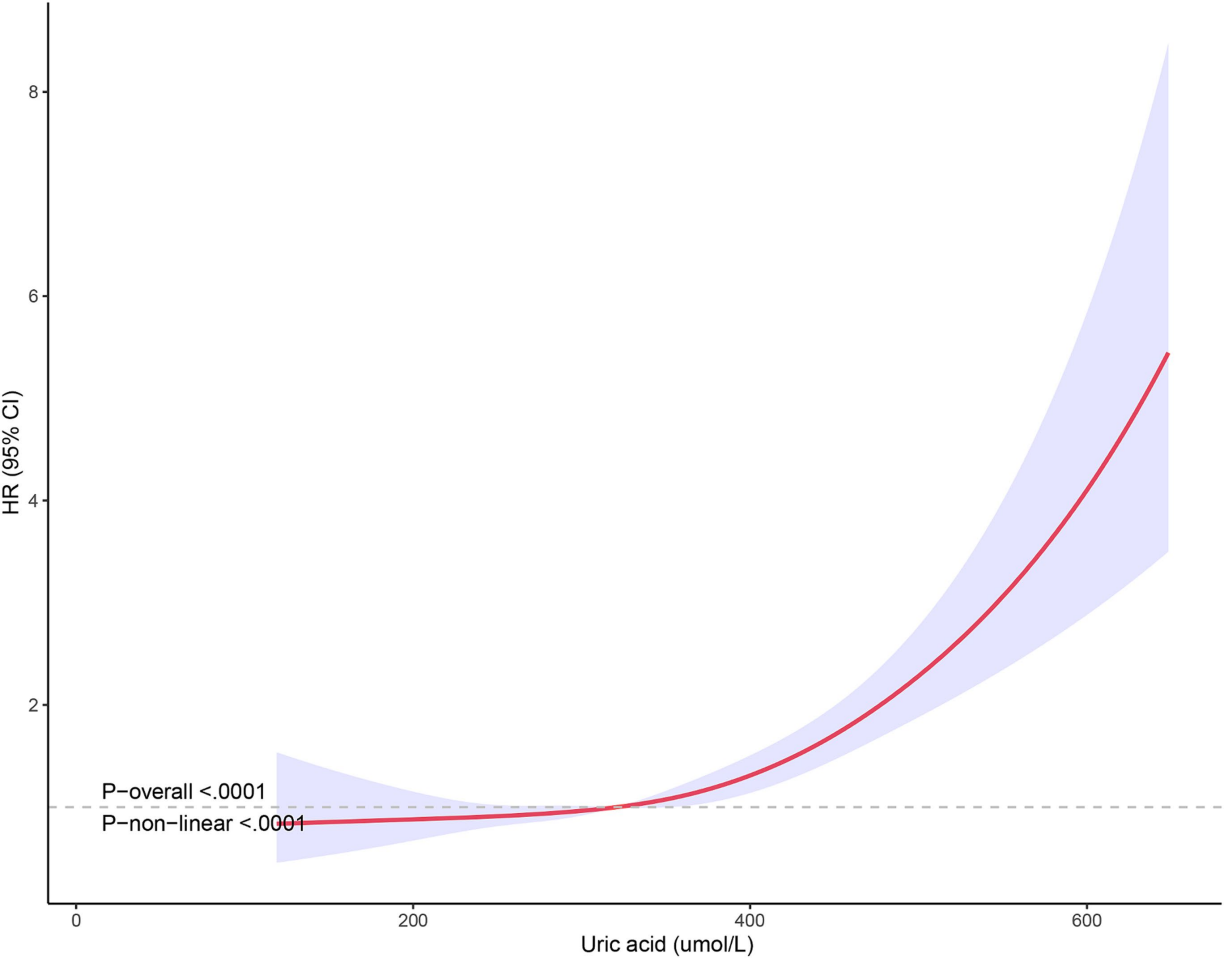
Nonlinear analysis of all-cause mortality and SUA ratio quintiles in female stroke patients.

**Figure 4:**
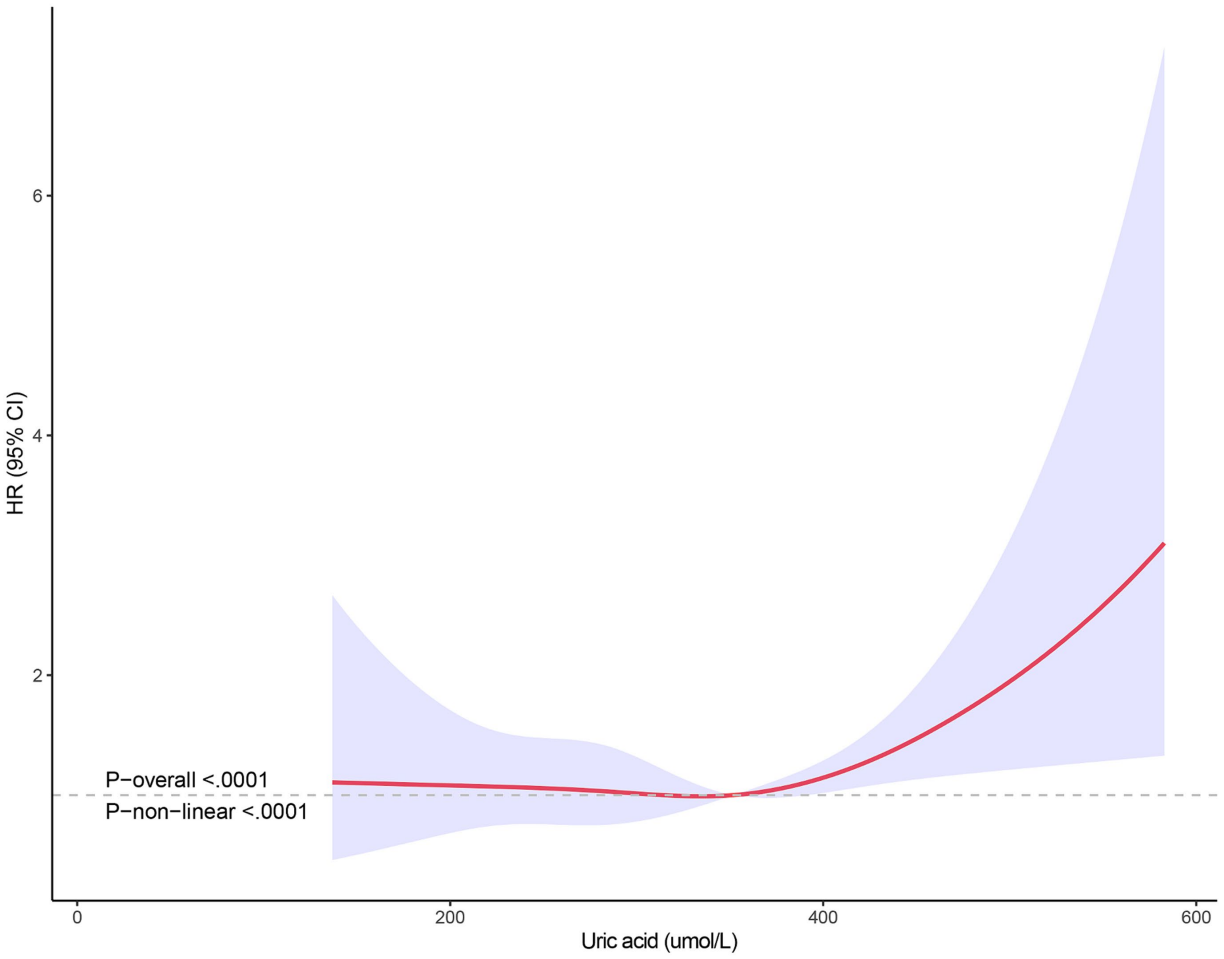
Nonlinear analysis of all-cause mortality and SUA ratio quintiles in male stroke patients.

The non-linear relationship between SUA levels and all-cause mortality was evaluated using RCS curves. Figure 2 illustrates the Cox-restricted cubic spline regression model employed for this purpose. The observed correlation between SUA levels and all-cause mortality exhibited a J-shaped curve (*p* < 0.0001) (Figure 2), thereby suggesting a non-linear interaction between these two variables. The Cox restricted cubic spline regression model depicted in Figure 3 showed the relationship between observed uric acid levels and all-cause mortality rate (*p* < 0.0001) for female patients with cerebral infarction, revealing a J-shaped curve indicative of a non-linear interaction between these variables. Additionally, Figure 4 presented the corresponding Cox restricted cubic spline regression model for male patients with cerebral infarction.

## Discussion

The findings of this study indicate a potential association between SUA levels and all-cause mortality in patients with stroke, notably in females. A high concentration of SUA correlates positively with all-cause mortality among these patients especially female patients, suggesting that SUA could serve as a promising biomarker for assessing the prognosis of patients afflicted with stroke. This correlation may offer novel perspectives on post-stroke treatment strategies.

The implementation of secondary prevention following a stroke can effectively mitigate the recurrence rate and mortality. However, there remains a subset of patients who experience stroke recurrence despite standard secondary prevention measures, with the underlying cause remaining unclear. The management of traditional risk factors such as hypertension, diabetes, and hyperlipidemia is pivotal in secondary prevention (19, 20). Notably, clinical diagnoses and treatments often reveal that some patients with poor prognosis do not exhibit these traditional risk factors associated with cerebrovascular disease. Consequently, proactively identifying potential risk factors for stroke recurrence and mortality holds significant importance for secondary prevention strategies. This area of research is currently a focal point in academic discourse.

SUA, as an antioxidant, plays a neuroprotective role in neurodegenerative diseases. However, its elevated levels can trigger inflammatory responses and exert oxidative effects. In a study of 3,731 patients with acute stroke, Weir et al. (21) discovered that higher SUA levels were an independent predictor of poor stroke prognosis and increased the likelihood of future vascular events. Conversely, a systematic evaluation and meta-analysis of 8,131 patients with acute stroke indicated that SUA levels in patients with favorable prognoses exceeded those in patients with poor prognoses, suggesting that SUA has a protective effect on neurological outcomes in these patients (22). This study further examines the impact of SUA on stroke-related mortality risk using the public NHANES database. Our findings corroborate a significant positive association between SUA and all-cause mortality in stroke patients, particularly among females and those under 60 years of age.

SUA typically circulates in the form of urate and is excreted in the urine. However, when kidney function and GFR is compromised, urate cannot be effectively excreted, leading to hyperuricemia (23). SUA exhibits both antioxidative and prooxidative properties. As a potent antioxidant and scavenger of singlet oxygen radicals, it can contribute to approximately 60% of blood-free radical scavenging capacity. Conversely, it can also induce endothelial dysfunction due to oxidative stress, resulting in inflammation and the release of ROS. This can lead to cardiovascular diseases such as stroke (24).

Currently, the relationship between SUA levels and the incidence and outcome of stroke remains a topic of debate. Zhang et al. (25) discovered no significant correlation between SUA levels and the prognosis of ischemic stroke. However, some researchers argue that patients with elevated SUA levels at the onset of stroke tend to have favorable clinical outcomes. Conversely, Sun et al. (26) found that increased SUA levels were positively associated with improved discharge recovery and a three-month prognosis in patients undergoing thrombolytic therapy for ischemic stroke. It has been established that SUA treatment in stroke patients treated with recombinant tissue plasminogen activator (rt-PA) is safe, reduces oxidative stress marker levels, and prevents premature SUA decline (27, 28). A 2021 meta-analysis demonstrated that SUA enhanced the prognosis of ischemic stroke by diminishing infarct size, bolstering the integrity of the blood-cerebrospinal fluid barrier, and ameliorating neurological function status in rodent studies (29). Conversely, Tu et al. (30) posited that patients with asymptomatic hyperuricemia exhibited a twofold elevated risk of stroke within a three-year span and served as an efficacious predictor of such events. A subsequent 2017 meta-analysis revealed a dose–response correlation between heightened SUA levels and stroke susceptibility, indicating an approximate 10% surge in stroke risk for every 1 mg/dL increment in SUA concentrations (31). A 2019 review, encompassing 33,580 stroke patients and 1,100,888 participants, similarly failed to provide substantial evidence supporting the hypothesis that SUA exerts a neuroprotective effect in ischemic stroke conditions (32). Consequently, the authors propose that elevated SUA levels may contribute to an increased incidence of stroke. A 2020 case-control study not only substantiated the notion that hyperuricemia is an independent risk factor for ischemic stroke, but also discovered that the severity of hypertension may moderate the impact of hyperuricemia on stroke. This research underscores that the synergistic effect of hyperuricemia and hypertension severity, coupled with treatment resistance, escalates the risk of ischemic stroke (33). The likelihood of stroke events correlates with both the cumulative exposure to SUA and its temporal progression. Early accumulation of SUA results in a higher risk compared to subsequent accumulation, underscoring the necessity of early control of SUA to optimal levels (34).

The J-shaped correlation with all-cause mortality was notably pronounced among females diagnosed with stroke. The influence of SUA on the prognosis of this condition is more evident in females, potentially attributable to the impact of estrogen. Estrogen facilitates the renal excretion of uric acid through a series of gene-level regulatory mechanisms. However, post-menopausal female lose the regulatory effect of estrogen on their uric acid levels.

### Limitation

This study presents several limitations. Firstly, the cross-sectional nature of our research precludes us from establishing causality; therefore, further prospective cohort studies are necessary to corroborate this finding. Secondly, the correlation between SUA and all-cause mortality in patients with stroke demonstrated variability based on sex and the presence or absence of hypertension. The interpretation of these results remains ambiguous and warrants additional exploration. Thirdly, the survival rate of patients can be influenced by antihypertensive drugs, anti-diabetic drugs, and lipid-lowering drugs. However, due to limitations in collecting drug information, we were unable to conduct a detailed subgroup analysis. This article solely focuses on the impact of uric acid on the overall population and further comprehensive research is warranted. Lastly, we acknowledge that there may be other unadjusted confounders influencing our findings, which could potentially introduce biases. These biases could potentially compromise the precision of true association assessments.

## Conclusion

In conclusion, our research indicates a potential association between SUA levels and all-cause mortality in patients with stroke, predominantly observed in females. SUA may serve as a promising biomarker for assessing the prognosis of these patients. This correlation could offer fresh perspectives on stroke treatment strategies, and further investigation is required to validate these observations.

## Data availability statement

The original contributions presented in the study are included in the article/supplementary material, further inquiries can be directed to the corresponding author.

## Author contributions

XT: Conceptualization, Data curation, Formal analysis, Investigation, Methodology, Project administration, Software, Supervision, Validation, Writing – original draft, Writing – review & editing. CL: Conceptualization, Data curation, Formal analysis, Methodology, Writing – original draft, Writing – review & editing. MG: Conceptualization, Data curation, Writing – review & editing. JG: Conceptualization, Data curation, Writing – review & editing. YZ: Funding acquisition, Methodology, Resources, Writing – original draft, Writing – review & editing.

## Footnotes

^1^ http://www.cdc.gov/nchs/nhanes.htm
